# Emergence of plasmid-borne *bla*_oxa-181_ gene in *Ochrobactrum intermedium*: first report from India

**DOI:** 10.1099/acmi.0.000024

**Published:** 2019-05-15

**Authors:** Thamaraiselvan Shanthini, Prasanth Manohar, Sagadevan Samna, Rajendran Srividya, Bulent Bozdogan, Manian Rameshpathy, Nachimuthu Ramesh

**Affiliations:** 1 Antibiotic Resistance and Phage Therapy Laboratory, Department of Biomedical Sciences, School of Bioscience and Technology, Vellore Institute of Technology (VIT), Vellore, Tamil Nadu, India; 2 Department of Microbiology, Recombinant DNA and Protein Research Center (REDPROM), Adnan Menderes University, 09100 Aydın, Turkey; 3 Department of Biotechnology, School of Biosciences and Technology, Vellore Institute of Technology (VIT), Vellore, Tamil Nadu, India

**Keywords:** *Ochrobactrum intermedium*, carbapenem-resistant, multi-drug resistance, carbapenemase, OXA, Gram-negative bacteria

## Abstract

Wastewater has become a potential habitat for multi-drug-resistant bacteria. The present study aims to screen for the presence of carbapenem-resistant bacteria in sewage water samples collected from hospital and non-hospital sources. From a total of 19 sewage water samples collected, 100 carbapenem-resistant non-lactose-fermenting Gram-negative bacteria (CR-NF-GNB) were isolated using MacConkey agar cultured with 8 mg l^−1^ of meropenem. On screening for beta-lactamase resistance genes (*bla*_NDM_, *bla*_OXA-48-like_, *bla*_IMP_, *bla*_VIM_ and *bla*_KPC_), one isolate, *Ochrobactrum intermedium *, was found to carry the plasmid-borne *bla*_OXA-48-like_ gene. To the best of our knowledge, we provide the first report of the rare and emerging opportunistic pathogen *Ochrobactrum intermedium* encoding the OXA-181 gene in its plasmid.

## Introduction

Antibiotics are extensively utilized for the treatment of infections caused by pathogenic bacteria in both humans and animals. The emergence of antibiotic-resistant bacteria is a serious global health problem. Further, there has been a proliferation of reports on carbapenem-resistant bacteria seeded in the environment in which the wastewater from hospitals plays a major role in the dissemination of resistance [1, 2]. *Acinetobacter baumannii *, *Enterobacter* spp., *Enterococcus faecium *, *Klebsiella pneumoniae *, *Pseudomonas aeruginosa* and *Staphylococcus aureus* have been highlighted as critical antibiotic-resistant bacteria by the Infectious Diseases Society of America, with Gram-negative bacteria being prominent [3]. Non-lactose-fermenting bacteria such as *A. baumannii *, *Burkholderia cepcia*, *P. aeruginosa *, *Stenotrophomonas maltophillia* and the recently emerging *Ochrobactrum* spp. are known to cause serious illness, especially in immunocompromised patients [4–6]. In the recent past, there have been reports on the emergence of antibiotic-resistant *Ochrobactrum* spp. from humans causing infections such as bacteraemia, endophthalmitis and a pyogenic liver abscess [7, 8]. In general, *Ochrobactrum* spp. has been known as an environmental pathogen with low virulence, but it has the potential to cause human infections [9]. It is evident that non-hospital environments have been found to play a major role in disseminating the antibiotic resistance genes, with this mainly occurring through horizontal gene transfer, while in some cases, mobile genetic elements such as integrons aid them in gaining resistance [10]. It is evident that antibiotic resistance genes are widespread and this is effectuated through the discharge of antibiotics and resistance genes to the environment, and the utilization of antibiotics in aquaculture, veterinary medicine and agricultural lands [11, 12]. This indicates that our battle against the spread of antibiotic resistance has not been waged adequately. In this study, we aimed to screen for the presence of carbapenem-resistant non-lactose-fermenting Gram-negative bacteria (CR-NF-GNB) from sewage water samples.

## Methods

### Sample collection

The sewage water samples were collected from various regions in Tamil Nadu during the period from August 2017 to January 2018. A total of 19 sewage water samples were collected in sterile containers and were immediately transported to the Antibiotic Resistance and Phage Therapy Laboratory, Vellore Institute of Technology, Vellore. Among the 19 samples, 7 were collected from hospital sewage and 12 samples were from non-hospital sources, including the river (*n*=2), the lake (*n*=1) and household sewage (*n*=9). Detailed sample collection and site information are given in Table 1.

**Table 1.** Sampling site information

Sample no.	Sampling location	No. of sewage samples collected from hospital outlet	No. of isolates recovered from hospital outlet	No. of sewage samples collected from non-hospital sewage	No. of isolates recovered from non-hospital sewage
1	Chennai	2	14+no colonies	1	10
2	Coimbatore	1	4	3	9+no colonies+no colonies
3	Erode	–	–	2	4+11
4	Madurai	1	5	–	–
5	Nilgiris	1	No colonies	2	No colonies
6	Pudukkottai	1	5	1	6
7	Salem	–	–	1	–
8	Trichy	1	11	1	14
9	Vellore	–	–	1	7
	**Total**	**7**	**39** **[39 %]**	**12**	**61** **[61 %]**

### Selective isolation of non- lactose fermenting Gram-negative bacteria (NF-GNB)

With a focus on isolating carbapenem-resistant non-lactose-fermenting Gram-negative bacteria, the collected sewage water samples were cultured on MacConkey agar (Himedia, India) with meropenem (8 mg l^−1^). The sewage water from different regions was spread-plated individually and the plates were incubated at 37 °C for 24 to 48 h.

### Minimum inhibitory concentration (MIC)

Once the non-lactose-fermenting Gram-negative bacteria from the sewage water were isolated, the MIC was determined for meropenem using the agar dilution method according to the Clinical and Laboratory Standards Institute (CLSI) guidelines (2018) [13]. In brief, Mueller–Hinton agar (MHA) plates were cultured with twofold increasing concentrations of meropenem and were dispensed from 0.25 mg l^−1^ to 128 mg l^−1^. The bacterial suspension was adjusted to 0.5 McFarland turbidity and 5 µl was spotted onto the plates. The plates were dried and incubated at 37 °C for 18 h. Growth shows the bacteria to be resistant and all the results were interpreted based on CLSI guidelines [13].

### DNA extraction and screening of carbapenem resistance genes

DNA was extracted using the boiling method as described previously [14]. Briefly, the isolated bacterial colony was homogenized in 150 µl of sterile distilled water and boiled at 100 °C for 20 min. Further, the samples were centrifuged at 10 000 ***g*** for 5 min and the supernatant was used as template DNA for polymerase chain reaction (PCR). Subsequently, the plasmid DNA was isolated using the HiPurA Plasmid DNA Miniprep Purification kit (Himedia, India). Chromosomal DNA contamination was checked using 16S rRNA primers as described previously [15]. Further, multiplex PCR was performed for the carbapenem resistance genes, *bla*_NDM_, *bla*_OXA-48-like_, *bla*_IMP_, *bla*_VIM_ and *bla*_KPC_ [14]. The isolate that carried the *bla*_OXA-48-like_ gene was identified by 16S rRNA analysis using the universal primers 27F and 1492R [14]. The amplified PCR product was sequenced (Eurofins Genomics Pvt Ltd, Bangaluru) and sequence analysis was performed using the blastn tool.

## Results

### Selection and screening of carbapenem-resistant non-fermenting Gram-negative bacteria

The sewage water samples (*n*=19) were cultured on meropenem (8 mg l^−1^) containing MacConkey agar plates. Bacterial growth did not appear within 24 h and the samples were incubated for a longer time. At 48 h, bacterial colonies were grown, and the non-lactose-fermenting colonies were chosen for further studies. Further, the colonies were purified on MacConkey agar supplemented with the 8 mg l^−1^ of meropenem. A total of 100 non-lactose-fermenting colonies were observed, including from hospital sewage (*n*=39) and non-hospital sources (*n*=61), including the river (*n*=10), the lake (*n*=0) and households (*n*=51). The MIC results showed that all the isolates were resistant to meropenem, and the MIC_50_ and MIC_90_ were found to be 64 mg l^−1^.

### Presence of the *bla*_OXA-48-like_ gene in *Ochrobactrum intermedium*

A total of 100 isolates were subjected to carbapenem resistance gene screening, and the beta-lactam gene, namely *bla*_OXA-48-like_, was found to be present in 1/100 non-lactose-fermenting Gram-negative bacteria. Interestingly, this isolate carried the resistance gene in its plasmid DNA. Genes such as *bla*_NDM_ *, bla*_IMP_, *bla*_VIM_ and *bla*_KPC_ were not present. The isolate that was found to have the *bla*_OXA-48-like_ gene was subjected to 16S rRNA analysis and found to be *O. intermedium *. Sequencing and an analysis report confirmed that the amplified OXA-48-like gene was OXA-181. The NCBI accession number for 16S rRNA is MH891792 and that for the OXA-181 gene is MH899445.

## Discussion

In the present study, carbapenem-resistant non-fermenting Gram-negative bacteria (CR-NF-GNB) that had been isolated from hospital and non-hospital sewage samples were extensively studied. There are limited studies on the prevalence of NF-GNB in the environment and the dissemination of carbapenem resistance among rare environmental pathogens requires attention. Among the studied NF-GNB (*n*=100), 39 % were isolated from hospital sewage and the remaining 61 % were isolated from non-hospital sources. In our study, the majority of NF-GNB were isolated from non-hospital sources, which is in contrast to the findings of an earlier study [16]. There are numerous reports in India on the distribution of carbapenem-resistant bacteria in environmental samples, with carbapenem-resistant *Enterobacteriaceae* from wastewater being much reported [17–20]. In this study, NF-GNB isolated from sewage water samples were found to be resistant to meropenem and the fact that the majority of the bacterial isolates were obtained from non-hospital sources shows the pervasiveness of carbapenem resistance among NF-GNB in India. Wastewater, and sewage water in particular, is becoming a reservoir for multi-drug-resistant bacteria and is promoting resistance among bacteria in the environment, with *Ochrobactrum* species serving as a good example of this.

*O. intermedium* is an emerging environmental human pathogen. Recently, there has been an increasing rate of infection caused by *O. intermedium *, with infection having been uncommon previously [7, 21, 22]. It was reported that most of the *O. intermedium* are multi-drug resistant but susceptible to carbapenem [7]. However, in our study, it was found that the *O. intermedium* isolated from hospital sewage samples was highly resistant to meropenem. At present, there is a high incidence of *Ochrobactrum* species becoming resistant to the beta-lactam class of antibiotics [7, 21, 22]. The evolution of drug resistance occurs chiefly by virtue of selection pressure and is enhanced by horizontal gene transfer. OXA-48-type beta-lactamase is predominantly encoded in the family *Enterobacteriaceae *, but in this study, *O. intermedium* was found to carry the plasmid-borne OXA-48-like gene belonging to the OXA-181 variant. There are reports on the prevalence of carbapenemase-producing *Enterobacteriaceae* predominantly carrying OXA-48 producers in wastewater [23]. To the best of our knowledge, this is the first study to report the presence of the OXA-48-type gene in *O. intermedium *. The dissemination of carbapenem-resistant bacteria in environmental samples is a serious health-care problem. Potent sewage treatment plants are not adequate to reduce this growing problem and effective surveillance programmes are necessary to monitor the spread the antibiotic-resistant bacteria.

### Conclusion

Our study shows that the sewage system acts as a potential hotspot for the proliferation of multi-drug-resistant bacteria and is an important source for the spread of resistance into the environment. The increase in the prevalence of carbapenem-resistant bacteria in the environment is creating an emergency. Hence, it is necessary to take immediate environmental monitoring steps in order to avoid the spread and emergence of antibiotic resistance among non-pathogenic bacteria present in the environment.
